# Whole‐genome sequencing of cell‐free DNA reveals DNA of tumor origin in plasma from patients with colorectal adenomas

**DOI:** 10.1002/1878-0261.13803

**Published:** 2025-01-20

**Authors:** Amanda Frydendahl, Adam J. Widman, Nadia Øgaard, Anushri Arora, Daniel Halmos, Jesper Nors, Johanne Ahrenfeldt, Tenna V. Henriksen, Christina Demuth, Line Raaby, Mads H. Rasmussen, Christina Therkildsen, Dan A. Landau, Claus L. Andersen

**Affiliations:** ^1^ Department of Molecular Medicine Aarhus University Hospital Denmark; ^2^ Department of Clinical Medicine Aarhus University Denmark; ^3^ Memorial Sloan Kettering Cancer Center New York NY USA; ^4^ New York Genome Center New York NY USA; ^5^ Weill Cornell Medicine New York NY USA; ^6^ Department of Pathology Aarhus University Hospital Denmark; ^7^ Gastro Unit, Copenhagen University Hospital Amager – Hvidovre Hospital Hvidovre Denmark

**Keywords:** adenomas, circulating tumor DNA, colorectal cancer, early detection, whole‐genome sequencing

## Abstract

The presence of circulating tumor DNA (ctDNA) in patients with colorectal adenomas remains uncertain. Studies using tumor‐agnostic approaches report ctDNA in 10–15% of patients, though with uncertainty as to whether the signal originates from the adenoma. To obtain an accurate estimate of the proportion of patients with ctDNA, a sensitive tumor‐informed strategy is preferred, as it ensures the detected signal originates from the adenoma. Here, tumor‐informed whole‐genome sequencing‐based ctDNA analysis (MRD‐EDGE^SNV^) was applied to two independent cohorts. Cohort 1, comprising 93 patients with stage III colorectal cancer (CRC) and 40 healthy individuals, was used to establish the signal threshold at 95% specificity. This threshold was then applied to Cohort 2, consisting of 22 patients with symptomatic and 20 with asymptomatic adenomas. In stage III, MRD‐EDGE^SNV^ had an area under the curve of 0.98. ctDNA was detected in 50% and 25% of patients with symptomatic and asymptomatic adenomas, respectively. The median adenoma plasma tumor fraction was 5.9 × 10^−5^. These finding not only demonstrate the feasibility of ctDNA detection in patients with colorectal adenomas, but also provides an estimate of the necessary sensitivity required to detect these lesions, paving the way for future ctDNA‐based screening strategies.

## Graphical abstract

Tumor‐informed whole‐genome sequencing (MRD‐EDGE^SNV^) was applied to detect circulating tumor DNA (ctDNA) in patients with colorectal adenomas. Using a 95% specificity threshold, established from stage III colorectal cancer patients, ctDNA was detected in 50% of symptomatic and 25% of asymptomatic adenoma cases with median tumor fractions of 5.9 × 10^−5^ and 7.8 × 10^−6^, respectively. This highlights the feasibility of ctDNA for adenoma detection.

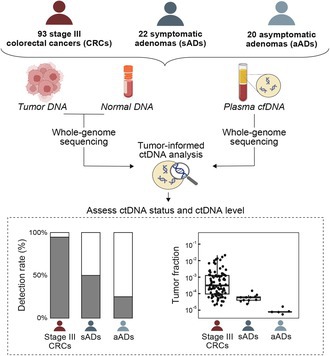

**Abbreviations**
aADs: asymptomatic adenomas
cfDNA: cell‐free DNA
CI: confidence interval
CRC: colorectal cancer
ctDNA: circulating tumor DNA
FIT: fecal immunochemical test
IQR: interquartile range
LOD: limit of detection
pre‐OP: pre‐operative
sADs: symptomatic adenomas
SNVs: single‐nucleotide variants
TF: tumor fraction
UICC: Union for International Cancer Control
VAF: variant allele frequency
WBCs: white blood cells
WGS: whole‐genome sequencing

## 1 Introduction

Circulating tumor DNA (ctDNA) has emerged as a promising biomarker for the detection and surveillance of cancer [1, 2]. Advancements in ctDNA detection techniques have significantly improved the ability to detect ctDNA, even in clinical settings where tumor burdens are low and shedding of tumor DNA is limited [3, 4, 5, 6]. This has paved the way for ctDNA to be explored as a tool for early cancer detection and, if possible, also for the detection of precancerous lesions, such as colorectal adenomas. Removal of adenomas has been reported as an effective way to reduce colorectal cancer (CRC) incidence [7, 8]. Therefore, it is relevant to understand whether ctDNA analysis has the potential to identify patients with adenomas. Previous studies investigating ctDNA detection from colorectal adenomas have reported a wide range of sensitivities and specificities [9, 10, 11, 12, 13, 14, 15], possibly due to the use of tumor‐agnostic detection methods, which may introduce uncertainty regarding the origin of the detected signal. Consequently, there is an incomplete understanding of ctDNA release from these lesions and an unmet need to know which limit of detection (LOD) is required to enable the detection of colorectal adenomas through ctDNA analysis. To obtain a more accurate estimate of ctDNA shedding from colorectal adenomas, tumor‐informed methods are essential. These methods ensure that the detected signal truly originates from the adenoma, mitigating potential interference from non‐neoplastic release of DNA with the same or similar epigenetic tags as those markers used by tumor‐agnostic methods. We recently introduced MRD‐EDGE^SNV^, a novel tumor‐informed approach that leverages the power of whole‐genome sequencing (WGS) and machine learning to achieve highly sensitive ctDNA detection [16]. This tumor‐informed approach has exhibited remarkable sensitivity in detecting ctDNA from colorectal lesions while upholding a high specificity [16], thus providing a unique opportunity to assess ctDNA at extremely low levels. Here, we applied the approach to quantify the ctDNA level in plasma from patients with symptomatic and asymptomatic colorectal adenomas.

## 2 Materials and methods

### 2.1 Study population

This study included 135 patients treated at seven Danish hospitals between November 2002 and February 2019, including 93 with UICC stage III CRC, 22 with symptomatic colorectal adenomas (sADs), and 20 with asymptomatic colorectal adenomas (aADs). Patients were categorized as symptomatic if their adenoma was diagnosed because they were referred for colonoscopy due to symptoms of colorectal cancer, or as asymptomatic if their adenoma was diagnosed due to participation in the Danish national population‐wide CRC screening program. In Denmark, CRC screening with a fecal immunochemical test is offered biannually to asymptomatic and average risk individuals aged between 50 and 74. The symptomatic patients were recruited either the day before or on the day of their colonoscopy and the asymptomatic patients in the period between the positive FIT test and the colonoscopy. Plasma samples were collected either the day before or on the day of colonoscopy. Tumor tissue from the removed adenoma was available from all patients.

The diagnostic procedure for patients with adenomas followed the Danish national guideline for pathoanatomical examination of colorectal polyps. Despite a large size of most adenomas, the pathoanatomical examination provided no indications suggesting invasive cancer.

Plasma samples from 40 healthy individuals without known cancer or adenomas were collected anonymously to serve as controls. Of these, 10 were obtained through the blood bank at Aarhus University Hospital, and the remaining 30 were sourced from the Colorectal Cancer Research Biobank at Aarhus University Hospital. The latter group comprised FIT‐positive patients without abnormal findings on subsequent colonoscopy. The study design, recruitment, and methodologies were conducted in full compliance with the ethical principles outlined in the Declaration of Helsinki. All participants provided written informed consent according to the Declaration of Helsinki. Ethics approval was obtained from the Danish National Committee on Health Research Ethics (case no. 2208092).

### 2.2 Sample collection and sequencing preparation

#### 2.2.1 Tumor and normal samples

Tissue specimens from stage III CRCs and sADs were obtained during colonoscopies conducted due to symptoms of colorectal illness. Tissue from aADs was collected from individuals who tested FIT‐positive as part of the National Danish CRC screening program and therefore got a diagnostic colonoscopy. White blood cells (WBCs) were collected from blood during plasma isolation. Tumor tissue and WBCs were stored at −80 °C until DNA extraction. Tumor tissue was collected either as fresh frozen (FF) or formalin‐fixed paraffin‐embedded (FFPE) tissue. A minimal tumor fraction of 20% (histological assessment) was required. DNA was extracted from FFPE tissue using QiAamp DNA FFPE tissue kit (Qiagen, Hilden, Germany), from FF tissue using the Puregene DNA Purification Kit (Gentra Systems, Minneapolis, MN, USA), and from WBCs using the QIAamp DNA Blood Kit (Qiagen). Tumor and normal DNA were quantified using the Qubit™ dsDNA BR Assay Kit (ThermoFisher, Waltham, MA, USA). Aliquoted tumor and normal DNA were shipped to New York Genome Center (NY, USA) where libraries were prepared and sequenced as previously described [16]. In brief, libraries were generated using the TruSeq DNA PCR‐Free Library Preparation Kit (FF and normal samples) or Kapa HyperPCR+ (FFPE samples) with minor modifications. DNA was concentration normalized and fragmented using a Covaris LE220 sonicator to a target size of 450 bp. After cleanup and end repair, an additional double‐sided bead‐based size selection was done to produce sequencing libraries with highly consistent insert sizes. This was followed by A‐tailing, ligation of Illumina DNA Adapter Plate adapters, and two postligation bead‐based library cleanups. Final libraries were run on a Fragment Analyzer (Agilent, Santa Clara, CA, USA) to assess their size distribution and quantified by qPCR with adapter‐specific primers (Kapa Biosystems, Wilminton, MA, USA).

#### 2.2.2 Plasma samples

Blood samples were collected in K2‐EDTA 10‐mL tubes (Becton Dickinson, Franklin Lakes, NJ, USA) prior to any treatment, that is, while the tumors were still in the bowel. Plasma was isolated within 2 h of blood collection by double centrifugation at 3000 ***g*** for 10 min and stored at −80 °C until DNA extraction. Circulating cell‐free DNA (cfDNA) was extracted using the QiaAMP Circulating DNA kit (Qiagen) and quantified using digital droplet PCR, as previously described [17]. Prior to cfDNA extraction, all plasma samples were spiked with a fixed number of soybean CPP1 DNA fragments, which was used to estimate purification efficiency, as previously described [18]. All sequencing libraries were generated in Aarhus using the KAPA HyperPrep kit (Roche, Bazel, Switzerland) using cfDNA from 2 mL of plasma or a minimum of 4 ng. cfDNA libraries were generated as previously described [19] using xGen UDI‐UMI Adapters (IDT, Coralville, IA, USA) and KAPA HyperPrep kit (Roche). Postligation cleanup was performed with AMPURE beads in a 1.4× (beads/DNA ratio) to retain short fragments, while post‐PCR cleanup was done using a 1.0× ratio. The libraries were amplified with seven cycles of PCR. Libraries were quantified using Qubit™ dsDNA BR Assay Kit (ThermoFisher) and the library fragment size was estimated using TapeStation D1000 (Agilent).

### 2.3 Whole‐genome sequencing

WGS was performed on tumor DNA, normal DNA from WBCs, and cfDNA from plasma samples using the NovaSeq platform (Illumina, San Diego, CA, USA) with paired‐end (2 × 150 bp) sequencing. Normal DNA and cfDNA were sequenced to a target coverage of 20×, while tumor DNA had target coverages of either 30× (fresh frozen biopsies) or 60× (formalin‐fixed paraffin‐embedded biopsies). A fraction of the sequencing data generated (48 stage III CRCs, 20 asymptomatic adenomas, and 40 healthy controls) was also included in another study [16].

### 2.4 Sample concordance

To identify potential inter‐patient sample swaps, we developed a strategy based on genotype concordance across approximately 1200 exonic single‐nucleotide polymorphisms (SNPs) with high genotype diversity. These 1200 sites were carefully selected to ensure uniform coverage as well as high sequencing and mapping quality across samples. Evaluation of the selected SNPs showed that they consistently could differentiate samples from the same patients and a random patient. To perform the sample concordance test, we extracted the genotypes of all 1200 SNPs from the mapped BAM files. Only SNPs with coverage above 10×, base quality above 10, and mapping quality above 10 were considered. Each SNP was genotyped using a maximum of 300 reads. Homozygous SNPs were identified by having an allele frequency greater than 0.85, while heterozygous SNPs fell within the allele frequency range of 0.35–0.65. SNPs outside these ranges were excluded from the analysis. The resulting “SNP score” represents the fraction (ranging from 0 to 1) of accepted SNP sites that exhibit concordance between the two samples being compared. For a patient's sample set to be considered coherent, the reference sample (WBC normal) must achieve SNP scores above 0.8 when compared to all other samples from the same patient, and SNP scores below 0.7 compared to representative samples (WBC normal) from all other patients.

### 2.5 ctDNA detection in plasma samples

For detection of ctDNA in plasma samples, we employed MRD‐EDGE^SNV^. The entire pipeline for MRD‐EDGE^SNV^, including preprocessing, variant calling, and use of deep learning model was conserved from prior work [16]. In brief, we established a compendium of high‐confidence somatic single‐nucleotide variants (SNVs) for each tumor as described previously [16]. To enable filtering of erroneous SNV calls caused by FFPE related noise, univariate Gaussian mixture models (GMM, sklearn.mixture) were used. For each FFPE tumor sample, a variant allele frequency (VAF) threshold was set, at the VAF where the GMM predict a 10% false‐positive rate. We only included SNVs with VAFs above this threshold. Detection of patient‐specific SNVs was performed by searching the plasma WGS for all sites from the matched tumor SNV compendium with corresponding mutations as described previously [16]. Artifactual and germline variants were filtered as described previously [16]. The MRD‐EDGE^SNV^ CRC classifier was trained and applied as described in prior work using plasma samples with high ctDNA levels from CRC patients and plasma from healthy controls [16]. The TF in plasma was calculated using the following equation

$$\mathrm{TF}=1-{\left(\frac{\left[M-\mu *R\right]}{N}\right)}^{\frac{1}{\mathrm{cov}}}$$

where *M* denotes the number of SNVs detected in the plasma sample, *N* denotes the number of SNVs (mutation load) in the patient‐specific mutational compendium, TF denotes the tumor fraction, cov denotes the local coverage in sites with a tumor‐specific SNV, μ denotes the noise rate (number of errors/number of reads evaluated) that corresponds to the patient‐specific SNV compendium, and *R* denotes the total number of reads covering the patient‐specific mutational compendium.

To address variation in sequencing artifact noise (μ) across patients with different mutational compendia, we applied a patient‐specific mutational compendium to calculate the expected noise distribution across the control plasma samples, as previously described [16]. The process described above was performed to detect the patient‐specific SNVs in control plasma samples. These detections represent the background noise model for which we calculated the mean and standard deviation (μ, σ) of artifactual mutation detection rate. Confident ctDNA detection can then be defined by converting the patient‐specific detection rate (det_rate = number of SNVs detected in cfDNA/number of reads checked = M/R) to a *Z* score = (det_rate − μ)/σ.

### 2.6 Determining *Z* score threshold for ctDNA detection

To determine a *Z* score threshold for ctDNA detection, each patient‐specific mutational compendium of the 93 stage III CRC patients was applied to the patient's own plasma sample (positive labels, *n* = 93) as well as each plasma samples from 40 healthy control (negative labels, *n* = 3720). The *Z* score threshold was defined as the threshold that kept the specificity above 95%. This threshold was applied to all plasma samples in the study.

### 2.7 Deep‐targeted sequencing of plasma samples

A subset of plasma samples (*n* = 87) from CRC patients were analyzed by deep sequencing of 16 patient‐specific targets using the Signatera™ assay (Natera, San Carlos, CA, USA). In brief, cfDNA was extracted from 8 mL plasma, and libraries were prepared using up to 66 ng of cfDNA and subjected to end‐repairing, A‐tailing and adapter liation, followed by amplification and purification. A 16‐plex targeted PCR was conducted on an aliquot of each library. Amplified, barcoded products were pooled and sequenced with an average > 100.000× raw coverage on an Illumina platform. A sample was called ctDNA positive if ≥ 2 variants were detected based on a previously defined confidence threshold [20]. The ctDNA level for each sample was calculated as the mean ctDNA level for all 16 targets, including targets without mutations detected.

### 2.8 Statistical analysis

Statistical analyses included Wilcoxon rank‐sum test or Fisher's exact test for unmatched groups, McNemar test and Cohen's Kappa for paired samples, and Pearson's correlation for linear correlation. Statistical calculations were done using R (v.4.2.0).

## 3 Results

### 3.1 Design

**Fig. 1:**
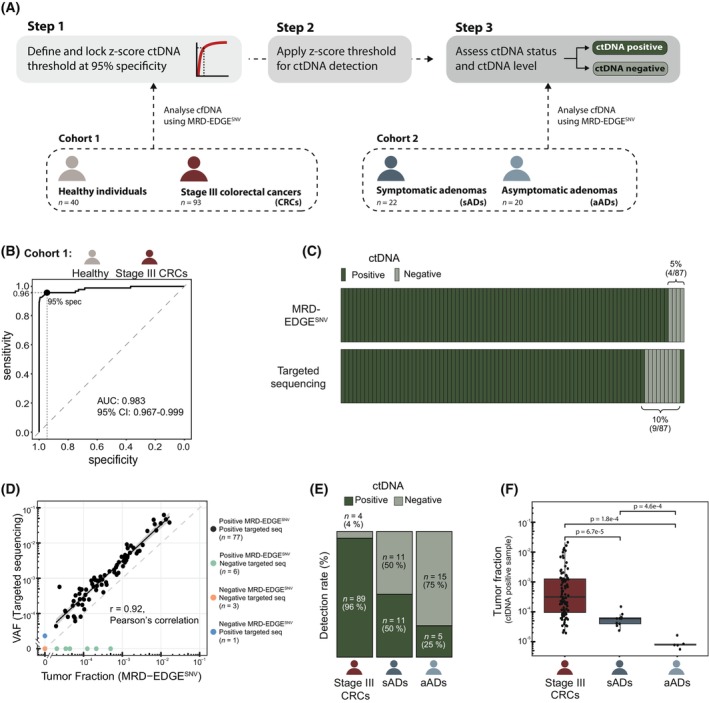
Study summary and MRD‐EDGE^SNV^ results. (A) Cohort 1 comprised 40 healthy controls and 93 patients with stage III colorectal cancers (CRC). Cohort 2 comprised patients with colorectal adenomas, including 22 symptomatic adenomas (sADs) and 20 asymptomatic adenomas (aADs). From all patients, tumor, normal, and pre‐operative plasma samples were subjected to whole‐genome sequencing. For healthy controls, a single plasma sample was used. Tumor‐informed circulating tumor DNA (ctDNA) analysis was performed using MRD‐EDGE^SNV^. Cohort 1 was used to determine a *z* score threshold for ctDNA detection, which was then applied to Cohort 2. (B) Receiver operating curve (ROC) analysis of pre‐operative (pre‐OP) plasma from patients with CRC (*n* = 93) and plasma from healthy controls (*n* = 40). Pre‐OP plasma samples (*n* = 93) were used as true labels, and false labels were each control plasma sample against all patients' mutational compendia (93 mutational compendia across 40 control samples, *n* = 3720). The dashed line indicates a random chance of ctDNA detection. (C) ctDNA detection status of plasma samples analyzed by MRD‐EDGE^SNV^ and tumor‐informed 16‐plex targeted sequencing (*n* = 87). Brackets denote the ctDNA‐negative samples identified by each respective method. (D) Comparison of estimated ctDNA levels estimated by MRD‐EDGE^SNV^ [tumor fractions (TFs)] and targeted sequencing [variant allele frequency (VAF)]. Linear regression includes samples called positive by both methods (black dots). The dashed gray line represents the line of equality, where TF and VAF values are identical. (E) Fraction of plasma samples called ctDNA‐positive or ctDNA‐negative, stratified by cohort. (F) MRD‐EDGE^SNV^ estimated TFs of ctDNA‐positive samples. Tumor fractions were compared between groups using the Wilcoxon rank‐sum test. Whiskers represent the smallest and largest value, excluding outliers.

We conducted tumor‐informed WGS‐based ctDNA analysis of two independent cohorts using MRD‐EDGE^SNV^. The first cohort (*n* = 93 stage III CRC, *n* = 40 healthy controls) was used to define a ctDNA detection threshold providing 95% specificity. The defined threshold was applied to the second cohort, which comprised 22 patients with symptomatic adenomas (sADs), and 20 patients with FIT‐screening detected asymptomatic adenomas (aADs) (Table 1). Figure 1A provides an overview of the study design.

**Table 1:** Patient characteristics.

Characteristic	*n*	Stage III CRC, *n* = 93^a^	Symptomatic adenoma, *n* = 22^a^	Asymptomatic adenoma, *n* = 20^a^
Gender	135			
Female		41 (44%)	15 (68%)	9 (45%)
Male		52 (56%)	7 (32%)	11 (55%)
Age	135	66 (60–72)	70 (64–80)	65 (57–67)
Tumor location	135			
Colon		83 (89%)	5 (23%)	14 (70%)
Rectum		10 (11%)	17 (77%)	6 (30%)
Tumor tissue	135			
FF		93 (100%)	22 (100%)	0 (0%)
FFPE		0 (0%)	0 (0%)	20 (100%)
Tumor size (mm)	114^b^	50 (38–70)	22 (14–39)	14 (10–21)
MMR status	135			
MSI		13 (14%)	0 (0%)	1 (5%)
MSS		80 (86%)	22 (100%)	19 (95%)
ctDNA status	135			
Negative		4 (4%)	11 (50%)	15 (75%)
Positive		89 (96%)	11 (50%)	5 (25%)

^a^ *n* (%); Median (IQR).

^b^ Tumor size not available for all tissue samples.

### 3.2 MRD‐EDGE^SNV^ is highly sensitive for plasma ctDNA in stage III CRC patients

We first applied MRD‐EDGE^SNV^ to pre‐operative (pre‐OP) plasma samples from patients with stage III CRC (*n* = 93) and plasma samples from healthy individuals (*n* = 40). This allowed us to assess the performance of MRD‐EDGE^SNV^ and establish a *z* score threshold for classifying a sample as ctDNA‐positive or negative. Receiver operating characteristic curve and precision–recall curve analysis demonstrated an exceptional performance of MRD‐EDGE^SNV^ with an area under the curve of 0.983 and 96% (89/93 ctDNA‐positive) sensitivity at 95% specificity (Fig. 1B, Fig. S1A). For a subgroup of patients (*n* = 87), we conducted a direct comparison between MRD‐EDGE^SNV^ and tumor‐informed 16‐plex deep targeted sequencing by analyzing 8 mL plasma samples obtained from the same blood collection. This revealed a strong concordance between the two methodologies, with 92% (80/87) of samples yielding the same classification (Fig. 1C). The sensitivity of MRD‐EDGE^SNV^ was 95% (83/87), while it was 90% (78/87) for deep‐targeted sequencing (Fig. 1C). When examining samples classified as ctDNA‐positive by both methods (*n* = 77), we observed a great correlation between the estimated ctDNA levels (*r* = 0.92 [95%‐CI: 0.88–0.95], *P* < 2.2e‐16, Pearson's correlation; Fig. 1D). The discrepancy in ctDNA status primarily occurred in samples with low tumor fractions (TF) (MRD‐EDGE^SNV^ TFs < 5 × 10^−4^; Fig. 1D).

### 3.3 ctDNA is detected in patients with colorectal adenomas

To evaluate the extent of ctDNA shedding from colorectal adenomas, we applied MRD‐EDGE^SNV^ to plasma samples from 22 sADs and 20 aADs using the *z* score threshold defined using plasma samples from stage III CRCs and healthy controls. A higher ctDNA detection rate was observed in plasma from sADs patients (50%, 11/22) than in plasma samples from aAD patients (25%, 5/20) (Fig. 1E). The median TFs in the plasma of patients with sADs and aADs were 5.9 × 10^−5^ (IQR: 4.0 × 10^−5^ – 6.3 × 10^−5^) and 7.8 × 10^−6^ (IQR: 7.6 × 10^−6^ – 8.4 × 10^−6^), respectively (Fig. 1F). This was significantly lower than the estimated TFs in plasma from CRC patients, which had a median TF of 3.1 × 10^−4^ (IQR: 9.5 × 10^−5^ − 1.3 × 10^−3^) (sADs: *P* = 6.7 × 10^−5^; aADs: *P* = 1.8 × 10^−4^; Wilcoxon rank sum) (Fig. 1F). Speculating, if adenoma size was a potential confounder of ctDNA detection, this was compared for aAD and sAD lesions. This revealed that sADs were larger than the aADs (*P* = 0.038) (Fig. S1B) and there was a trend toward larger adenomas among the ctDNA‐positive patients compared to the ctDNA‐negative patients within each group of adenomas (sADs: *P* = 0.2, aADs: *P* = 0.27) (Fig. S1C).

## 4 Discussion

To assess the ctDNA level in the plasma of patients with colorectal adenomas, a tumor‐informed strategy is essential, as it ensures that the detected signal indeed originates from the adenoma, thereby providing a more accurate estimate of the ctDNA level. In this study, we utilized the highly sensitive MRD‐EDGE^SNV^ approach [16] and defined a *Z*‐score threshold at which the specificity of the approach was 95%. Using this threshold, we demonstrated that ctDNA was detectable in up to 50% of colorectal adenomas. Robust detection of ctDNA in adenoma patients required an LOD of ~ 10^−5^ due to their extremely low ctDNA levels. We observed lower detection rates and ctDNA levels among aADs compared to sADs, possibly due to the smaller size of these lesions. Previous studies have reported a strong correlation between tumor size and ctDNA detection in CRC and other cancers [21, 22]. These studies also showed that the ctDNA levels in patients with large lesions are higher than in patients with small lesions. Accordingly, adenoma patients who initially are ctDNA negative may become positive as the lesion grows, providing an opportunity for early detection of the lesions through subsequent rounds of screening.

Detection of ctDNA from colorectal adenomas is promising, but our findings also demonstrate the major challenge of ctDNA‐based screening, namely achieving sufficient sensitivity to allow detection. Recent studies employing tumor‐agnostic approaches have reported adenoma detection rates around 10–15%, underscoring the challenge of these methods in achieving the necessary sensitivity for detecting such lesions [11, 15]. Given the results of these studies, it may be challenging to obtain high enough sensitivity of tumor‐agnostic approaches. However, an important question also arises: how early do we need to detect precancerous lesions to facilitate successful cancer prevention and treatment? Undeniably, early detection is key for effective cancer treatment [23], yet, identifying colorectal adenomas at a slightly later stage, when they are larger and release more ctDNA, may still allow for effective treatment. However, this warrants exploration through large, randomized studies. Nevertheless, the possibility of detecting ctDNA detection from precancerous lesions, although challenging, demonstrates the potential for blood‐based testing. Possibly, a combined approach using both blood‐based ctDNA testing and stool‐based FIT testing could help guide and prioritize individuals for colonoscopies or further diagnostic evaluations. Additionally, offering a ctDNA‐based approach to individuals who do not comply with stool‐based testing could significantly enhance participation rates in screening programs [24]. However, as not all adenomas progress into malignancies, the use of ctDNA‐based approaches should be balanced with the awareness to the risk overdiagnosis considering the potential psychological and clinical consequences of identifying lesions that may never pose a significant threat.

MRD‐EDGE^SNV^ is a tumor‐informed approach, and hence not suitable as a screening tool. Nonetheless, the objective of our study was not to develop a screening test but rather to evaluate the extent of ctDNA release from colorectal adenomas, and to estimate the LOD needed for a ctDNA test to be able to detect adenomas. It is worth noting that the estimated TF obtained through MRD‐EDGE^SNV^ consistently tends to be slightly lower than the VAF estimated by targeted sequencing. This is probably related to differences in the number of ctDNA markers and the criteria for selecting them. The targeted sequencing approach uses only 16 mutations and prioritizes mutations that are clonal and have high multiplicity. Accordingly, the mutations are intentionally chosen to be on the high end of the tumor VAF distribution. By contrast the extensive mutation compendium used in MRD‐EDGE^SNV^ includes also subclonal variants, which may lead to a slight underestimate of the TF. These assay‐specific differences in TF estimates should be carefully considered when comparing assay TF LODs.

A major strength of our study is the high specificity and sensitivity provided by the WGS based tumor‐informed approach. In the current study, the *Z*‐score threshold was calculated using all available training data. However, we have previously shown that the *Z*‐score threshold is highly robust and yields the same specificity performance in independent negative control cohorts, even when generated using different sequencing chemistries and sequencing platforms than the NovaSeq platform used for training [16]. This suggests that the *Z*‐score thresholds and negative control data can be reused from study to study.

While our study documents that adenoma patients do have ctDNA in their blood, we acknowledge several limitations, including a limited number of adenoma patients, focus on patients with large adenomas, and a systematic difference in the tumor profiling of sAD and aADs. The tumor tissue of the former was FF and of the latter FFPE. Sequencing of DNA from FFPE tissue is more error prone, which despite our sincerest bioinformatic efforts to avoid it may have caused the somatic tumor mutation compendiums of the aADs to contain erroneous mutations not present in the compendiums of the sADs, which potentially may have affected ctDNA detection in aADs. To better understand the factors influencing ctDNA shedding from adenomas, future studies should include larger and more diverse cohorts. Lastly, as our study focused on colorectal lesions, we do not provide insights into whether precancerous lesions in other organs also shed tumor DNA to the circulation.

## 5 Conclusions

In summary, MRD‐EDGE^SNV^ showed great performance in detecting ctDNA, as demonstrated by analysis of pre‐OP plasma samples from stage III CRC patients as well as comparison to targeted sequencing. Through tumor‐informed ctDNA analysis with MRD‐EDGE^SNV^, we demonstrate that ctDNA can be detected from patients with colorectal adenomas (50% of sADs, 25% of aADs). The observed TFs were below 1.5 × 10^−4^ in sADs and below 1.6 × 10^−5^ in aADs, which emphasize the need for highly sensitive detection methods. Given the observed TFs, achieving an LOD of approximately ~ 10^−5^ through tumor‐agnostic ctDNA analysis, along with acceptable specificity, holds promise for the potential viability of ctDNA‐based screening for precancerous lesions.

## Conflict of interest

DAL and AW submitted two patent applications. CLA reports collaborations with C2i Genomics and Natera. DAL received research support from Illumina, Inc. DAL is a scientific co‐founder of C2i Genomics.

## Author contributions

AF, AJW, DAL, and CLA designed the study. AF, NØ, and TVH were responsible for DNA extractions and sample preparation. JN, LR, CT, CD and NØ contributed to the collection of clinical data and selection of patients for the final study. AF and AW were responsible for library preparation and sequencing. AW, AA, and DH were responsible for running MRD‐EGDE^SNV^. AF, JA, AW, AA, DH, TVH, and MHR conducted data analysis. DL and CLA supervised and supported the study. AF drafted the manuscript, which was revised by all authors.

### Peer review

The peer review history for this article is available at https://www.webofscience.com/api/gateway/wos/peer‐review/10.1002/1878‐0261.13803.

## Supporting information


**Fig. S1.** Precision–recall curve and adenoma characteristics.

## Data Availability

To protect the privacy and confidentiality of patients in this study, personal data including clinical and sequence data are not made publicly available in a repository or the supplementary material of the article. The data can be requested from the corresponding author. Any requests will be reviewed within a time frame of 2–3 weeks by the data assessment committee to verify whether the request is subject to any intellectual property or confidentiality obligations. All data shared will be de‐identified. Access to clinical data and processed sequencing data output files used in the article requires that the data requestor (legal entity) enter into Collaboration and Data Processing Agreements, with the Central Denmark Region (the legal entity controlling and responsible for the data). Request for access to raw sequencing data furthermore requires that the purpose of the data re‐analysis is approved by The Danish National Committee on Health Research Ethics. Upon a reasonable request, the authors, on behalf of the Central Denmark Region, will enter into a collaboration with the data requestor to apply for approval.
