# Supplementary material for: Whole‐genome sequencing of cell‐free DNA reveals DNA of tumor origin in plasma from patients with colorectal adenomas
Source: Mol Oncol. 2025 Jan 20;19(4):984–93. doi: 10.1002/1878-0261.13803 (PMC11977638; doi:10.1002/1878-0261.13803)
Supplement: Supplementary file 1 — Fig. S1. Precision–recall curve and adenoma characteristics. [file MOL2-19-984-s001.zip › Figure_Legends.docx]

**Figure S1.** Precision–recall curve and adenoma characteristics. **A)** Precision–recall curve analysis of pre-operative (pre-OP) plasma from patients with colorectal cancer (CRC) (n = 93) and plasma from healthy controls (n = 40). Pre-OP plasma samples (n = 93) were used as true labels, and false labels were each control plasma sample against all patients' mutational compendia (93 mutational compendia across 40 control samples, n = 3720). **B)** Comparison of adenoma size (mm) among symptomatic and asymptomatic adenomas. Outliers not shown. **C)** Comparison of adenoma size among circulating tumor DNA (ctDNA)-positive and ctDNA-negative patients within each adenoma group. *Tumor size not available for all tissue samples.
