# Supplementary figures and images for: Whole‐genome sequencing of cell‐free DNA reveals DNA of tumor origin in plasma from patients with colorectal adenomas
Source: Mol Oncol. 2025 Jan 20;19(4):984–93. doi: 10.1002/1878-0261.13803 (PMC11977638; doi:10.1002/1878-0261.13803)

# SUPPLEMENTAL CONTENT - Figure 1

**A**

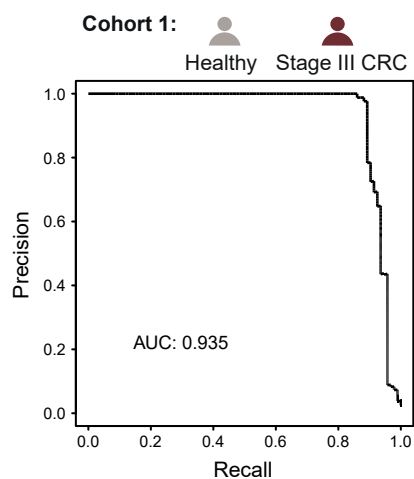

**B**

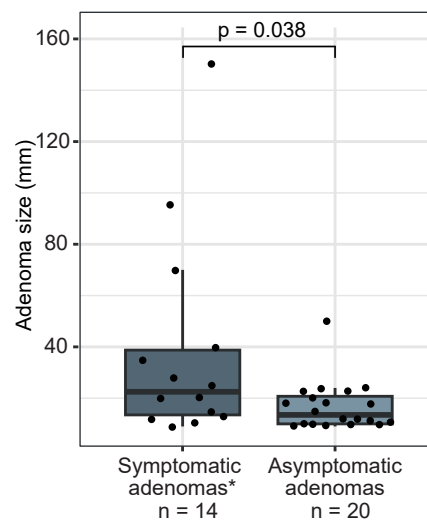

**C**

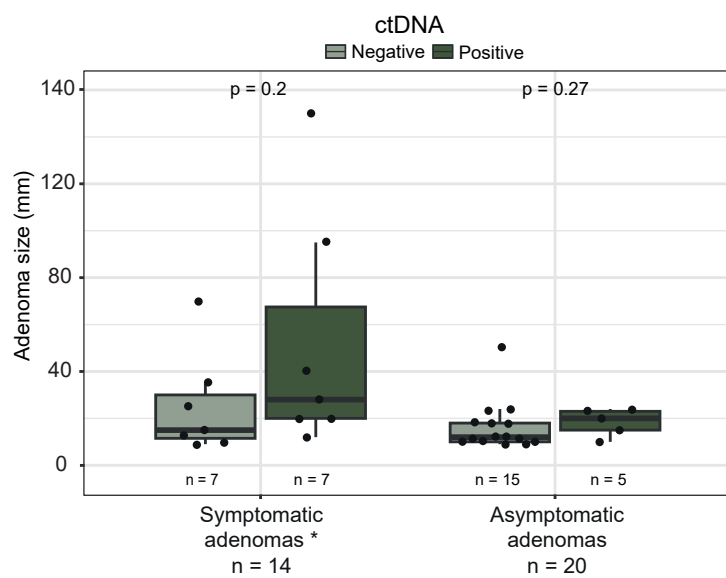

Supplement: Supplementary file 1 — Fig. S1. Precision–recall curve and adenoma characteristics. [file MOL2-19-984-s001.zip › mol213803-sup-0001-FigureS1.pdf]
